# Dr. Charles Herbert Davie

**Published:** 1908-07

**Authors:** 


Dr. Charles Herbert Davie, of South Scituate, Mass., died suddenly in the South Station, Boston, June 4, 1908, from heart disease, aged sixty-five years. He graduated at the University of Buffalo, medical department, in 1865.

